# MKK4 and MKK7 control degeneration of retinal ganglion cell somas and axons after glaucoma-relevant injury

**DOI:** 10.1038/s41420-025-02842-w

**Published:** 2025-12-15

**Authors:** Olivia J. Marola, Stephanie B. Syc-Mazurek, Sarah E. R. Yablonski, Peter G. Shrager, Simon W. M. John, Richard T. Libby

**Affiliations:** 1 The Jackson Laboratory, Bar Harbor, ME USA; 2 Department of Ophthalmology, University of Rochester Medical Center, Rochester, NY USA; 3 Departments of Neurology and Ophthalmology, Mayo Clinic, Rochester, MN USA; 4 Neuroscience Graduate Program, University of Rochester Medical Center, Rochester, NY USA; 5 Department of Neuroscience, University of Rochester, Rochester, NY USA; 6 Department of Ophthalmology, Vagelos College of Physicians and Surgeons, Zuckerman Mind Brain Behavior Institute, Columbia University, New York, NY USA; 7 The Center for Visual Sciences, University of Rochester, Rochester, NY USA; 8 Department of Biomedical Genetics, University of Rochester Medical Center, Rochester, NY USA

**Keywords:** Cell death in the nervous system, Apoptosis, Neurodegeneration

## Abstract

Retinal ganglion cell (RGC) death is a critical component of glaucoma pathology. The degenerative signaling pathways that lead to RGC death in glaucoma are incompletely defined. Recently, the transcription factors JUN and DDIT3 were identified as critical hubs regulating RGC somal loss after mechanical axonal injury. However, their position within the degenerative cascade remains unclear. One possibility is that JUN and DDIT3 activity in the soma initiates signaling events that trigger axonal degeneration. Alternatively, JUN and DDIT3 may function downstream of the primary insult, acting specifically to mediate somal degeneration without influencing axonal pathology. Disentangling these possibilities is critical for understanding the compartment-specific mechanisms of RGC degeneration in glaucoma. The MAP2Ks MKK4 and MKK7 control JNK and JUN activity and can indirectly activate DDIT3. Furthermore, MKK4 and MKK7 have been shown to drive RGC axonal degeneration after mechanical axonal injury. The present work investigated whether JUN and DDIT3, or their upstream activators MKK4 and MKK7, control degeneration of RGC axons and somas after glaucoma-relevant injuries; including ocular hypertension in aged DBA/2J mice and after mechanical axonal injury (controlled optic nerve crush, CONC) in C57BL/6J mice. *Ddit3* and *Jun* deletion did not prevent RGC axonal degeneration in DBA/2J mice but prevented nearly all somal loss. Despite robust somal survival, *Ddit3* and *Jun* deletion did not prevent RGC somal shrinkage or pattern electroretinography (PERG) amplitude decline in DBA/2J mice or after CONC in C57BL/6J mice. In contrast, *Mkk4* and *Mkk7* deletion from C57BL/6J mice significantly lessened RGC soma and axon degeneration while preserving PERG amplitude and soma size after CONC. In summary, activation of MKK4 and MKK7 may be an inciting mechanism governing RGC somal and axonal degeneration after glaucoma-relevant axonal injury.

## Introduction

Vision loss in glaucoma is caused by progressive loss of retinal ganglion cells (RGCs). One of the most important risk factors for developing glaucomatous neurodegeneration is elevated intraocular pressure (IOP) [1, 2]. Much progress has been made in dissecting the mechanisms of RGC death after glaucoma-relevant injury. Several human and animal studies have indicated that RGC axonal injury at the optic nerve head [3–8] and death of RGCs [9–14] are critical pathologies in glaucoma. Furthermore, several studies have suggested RGC somal and axonal degeneration are governed by distinct (though not necessarily fully separate) molecular mechanisms after axonal injury. For example, the pro-apoptotic molecule BAX was required for RGC somal death after glaucoma-relevant RGC axonal injury (controlled optic nerve crush, CONC [9, 15, 16]) and in ocular hypertensive DBA/2J mice [9, 10]. However, BAX did not contribute to RGC axonal degeneration in these models [3, 9, 10]. Subsequent studies have shown the mitogen activated protein kinases MAPK12 (DLK) and the Jun N-terminal Kinases MAPKs 9 and 10 (JNK2/3) drive RGC somal loss but not axonal degeneration after CONC [12, 17, 18]. Furthermore, overexpression of the pro-survival Bcl2 family protein BCLXL prevented RGC somal loss, but not axonal degeneration after CONC [19]. Manipulation of axonal signaling pathways with the *Wld*^*S*^ transgene or *Sarm1* deletion delayed axonal degeneration without protecting from somal degeneration after CONC [11–13, 20]. Given degeneration of the RGC soma and axon are driven by distinguishable mechanisms, identifying a mechanism triggering both RGC somal loss and axonal degeneration cascades will be critical for the identification of neuroprotective therapies in glaucoma.

Recently, the transcription factors JUN and DNA-damage inducible transcript 3 (DDIT3, also known as CHOP) were identified as critical regulators of RGC somal loss after glaucoma-relevant injury [14, 21–23]. *Ddit3* and *Jun* (*Ddit3/Jun*) deletion provided near-complete protection to RGC somas after CONC [21]. Like *Bax* [9]*, JNK2/3* [12], and *Dlk* [12] deletion, *Ddit3*/*Jun* deletion did not prevent RGC axonal degeneration after CONC [21], and individual deletions of *Ddit3* [22] or *Jun* [14] were not sufficient to prevent RGC axonal degeneration in DBA/2J mice. It is possible DDIT3 and JUN act together, playing redundant and compensatory roles to integrate somal and axonal degeneration cascades in the context of ocular hypertension. Here, we test the role of DDIT3 and JUN together in driving RGC somal and axonal degeneration in the DBA/2J model of chronic ocular hypertension.

Alternatively, it is possible that DDIT3/JUN act as critical downstream hubs regulating RGC somal loss but not axonal degeneration—which would suggest that a mechanism upstream of DDIT3 and JUN activation governs degeneration of both RGC axons and somas.. Therefore, we also investigated the importance of MAP2K4 and MAP2K7 (MKK4 and MKK7; MKK4/7). MKK4/7 are known to act upstream of JUN activation through the JNKs. While alternative or compensatory mechanisms have been reported to modify JNK activation in various cell types and stress conditions [24–26], MKK4/7 are the primary and best-characterized MAP2Ks upstream of the JNKs and are the only kinases known to directly phosphorylate JNKs [27–29]. MKK4 also activates JNK-independent MAPK11-14 (p38) [30]—which is known to directly activate DDIT3 [31] and has been suggested to play a role in loss of axonal transport and degeneration [32, 33]. Through the JNKs, MKK4/7 can control signaling mechanisms known to drive axonal degeneration, including BH3-only protein activation [34, 35], NMNAT2 degradation [36, 37], and SARM1 activation [38]. Therefore, MKK4/7 may serve as upstream activators of DDIT3, JUN, and DDIT3/JUN-independent axonal degeneration signaling cascades.

The roles of MKK4 and MKK7 have been tested in models of glaucoma-relevant injury. Individually, *Mkk4* or *Mkk7* deletion afforded significant but incomplete protection to RGC somas after CONC (42% and 17% protection, respectively) [39]. Neither *Mkk4* nor *Mkk7* deletion alone was sufficient to prevent all JNK and JUN activation in RGC axons and somas after CONC [39], suggesting at least partial compensatory activity. Excitingly, blocking both MKK4 and MKK7 activity preserved morphological integrity of axons [32, 38] including RGC axons [38], after axonal injury. Therefore, MKK4/7 signaling may be a critical early mechanism governing RGC somal death via DDIT3/JUN activation and may also regulate axonal degeneration after glaucoma-relevant injury. Here, we assessed the importance of DDIT3 and JUN, along with their upstream activators MKK4 and MKK7, in RGC degeneration after glaucoma-relevant injury.

## Results

### DDIT3 and JUN controlled RGC somal loss after ocular hypertension

DDIT3 and JUN together have been shown to regulate death of RGC somas after CONC, potentially additively [21]. It has yet to be determined whether somal DDIT3/JUN activity together initiates axonal degeneration mechanisms. To determine whether both JUN and DDIT3 act in tandem to elicit RGC axonal degeneration in the context of glaucoma, *Ddit3* and/or *Jun* were deleted from the full body and neural retina, respectively, from ocular hypertensive DBA/2J mice.

**Fig. 1:**
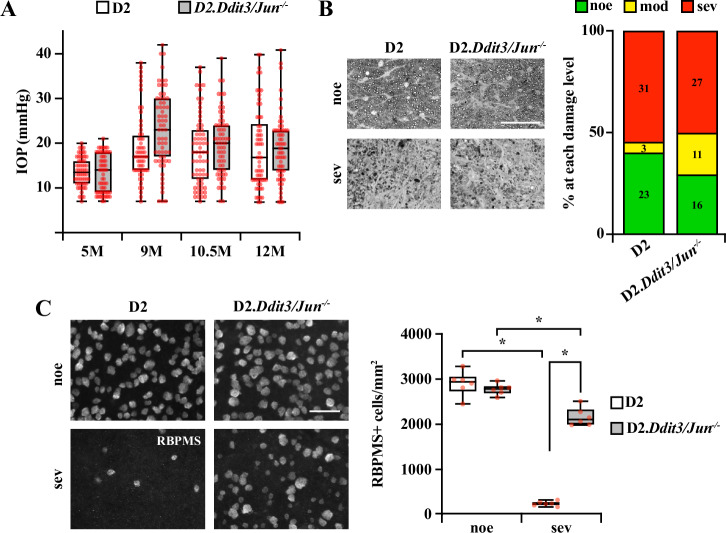
*Ddit3/Jun* deletion did not prevent axonal degeneration but robustly improved RGC somal survival in DBA/2 J mice. **A** Longitudinal intraocular pressure (IOP) measurements from D2 and D2.*Ddit3/Jun*^*−/−*^ eyes (*n* listed respectively here and throughout figure legends) at 5 M (*n* = 60, 63), 9 M (*n* = 60, 63), 10.5 M (*n* = 66, 63), and 12 M (*n* = 62, 61). Both genotype groups had significantly elevated IOPs at 9 M, 10.5 M, and 12 M compared to 5 M (*P < 0.001). At these timepoints, D2.*Ddit3/Jun*^*−/−*^ eyes did not have a statistically significant reduction in IOP compared to WT, although D2.*Ddit3/Jun*^*−/−*^ eyes had slightly higher IOPs at 9 M of age compared to D2 (*P = 0.004). Two-way ANOVA, Holm-Sidak’s *post hoc*. **B** Examples of optic nerve cross sections with no or early (noe) and severe (sev) glaucomatous damage from D2 and D2.*Ddit3/Jun*^*−/−*^ mice and percentages of optic nerves with noe (*n* = 23, 16), moderate (mod; *n* = 3, 11) and sev (*n* = 31, 27) glaucomatous damage. *Ddit3/Jun* deletion did not afford protection to RGC axons and in fact slightly worsened axonal degeneration (*P = 0.049). Chi square test. **C** Representative retinal flat mounts immunoassayed for RBPMS and quantification of RBPMS+ cells from 12 M D2 and D2.*Ddit3/Jun*^*−/−*^ retinas with corresponding noe (*n* = 6, 6) or sev (*n* = 6, 6) optic nerves. Sev D2.*Ddit3/Jun*^*−/−*^ retinas had 77.0 ± 3.1% improved RGC survival compared to D2 controls (*P < 0.001). RBPMS+ cells/mm^2^ ± SEM for D2 and D2.*Ddit3/Jun*^*−/−*^ respectively: Noe: 2899.6 ± 111.0, 2765.4 ± 49.8; Sev: 226.7 ± 20.8, 2151.5 ± 83.0. Two-way ANOVA, Holm-Sidak *post-hoc*. Scale bars, 50 μm.

Neurodegeneration in the DBA/2J model of glaucoma is dependent upon elevated IOP [40–45]. Some genetic manipulations have been reported to lower IOP in DBA/2J mice [46]. However, with Six3-cre, *Jun* is not deleted in the structures in the anterior segment of the eye that control IOP, and D2.Six3-cre^+^*Jun*^*fl/fl*^ mice did not have altered IOP compared to D2 controls [14]. Furthermore, full-body deletion of *Ddit3* did not alter the IOP profile of the DBA/2J mouse model [22]. To ensure combined *Ddit3/Jun* deletion did not lessen IOP elevation, IOPs were measured from D2.*Ddit3*^*+/?*^*Jun*^*+/?*^ (D2; WT) and D2.Six3-cre^+^*Ddit3*^*−/−*^*Jun*^*fl/fl*^ (D2.*Ddit3/Jun*^*−/−*^) mice at 5 M, 9 M, 10.5 M, and 12 M of age. Mice of both genotypes had elevated IOP at each timepoint compared to 5 M, and IOP was not lowered compared to WT D2 at each timepoint measured. Therefore, *Ddit3*/*Jun* deletion did not substantially alter the profile of ocular hypertension typical of the DBA/2J model (Fig. 1A).

To determine whether DDIT3 and JUN control death of RGC axons after ocular hypertension, D2.*Ddit3/Jun*^*−/−*^ and WT D2 control optic nerves were assessed for axonal degeneration at 12 M—a timepoint at which roughly 50% of D2 optic nerves will have severe optic nerve damage [47]. *Ddit3/Jun* deletion did not lessen instances of severe glaucomatous neurodegeneration. In fact, D2.*Ddit3/Jun*^*–/–*^ mice had slightly worse outcomes relative to D2 controls (Fig. 1B). Therefore, DDIT3 and JUN did not act in tandem to perpetuate axonal degeneration after chronic ocular hypertension in this model.

Several molecules contribute to degeneration of the soma, but not the axon, after glaucoma-relevant injury [9, 14, 22]. To determine whether DDIT3 and JUN play an important role in RGC somal degeneration after severe axonal injury, 12 M D2.*Ddit3/Jun*^*−/−*^ and D2 retinas with corresponding severe optic nerves were assessed for RGC somal survival. *Ddit3/Jun* deletion conferred robust (77%) protection to RGC somas in retinas with severe optic nerve degeneration (Fig. 1C). These data suggest DDIT3 and JUN are critical regulators of somal death but not axonal degeneration after chronic ocular hypertension.

### Despite somal survival, *Ddit3* and *Jun* deletion did not prevent PERG amplitude decline or somal shrinkage

Ocular hypertension is known to cause impaired RGC somal gross potentials [3, 48–51], even before the onset of detectable axonal damage [51, 52]. Others have shown neuroprotective treatment or genetic manipulation also preserved physiological activity in glaucoma-relevant models [3, 48–50]. Given *Ddit3*/*Jun* deletion protected most RGC somas after chronic ocular hypertension (Fig. 1C), it remained important to determine whether surviving D2.*Ddit3/Jun*^*−/−*^ RGC somas retained gross physiological function. To test this, pattern electroretinograms (PERGs) were longitudinally recorded from D2, D2.*Ddit3*^*−/−*^*Jun*^*+/?*^ (D2.*Ddit3*^*−/−*^), D2.Six3-cre^+^ *Ddit3*^*+/?*^*Jun*^*fl/fl*^ (D2.*Jun*^*−/−*^), and D2.*Ddit3/Jun*^*−/−*^ animals at 5 M (before the onset of IOP elevation and RGC degeneration [47]), 9 M (when IOP is elevated, but morphologically detectable RGC degeneration has not yet occurred [47]), and 12 M of age (when roughly 50% of eyes have severe glaucomatous neurodegeneration [47], Fig. 1B). IOP elevation in DBA/2J mice is dependent upon a loss-of-function point mutation in *Gpnmb*; therefore, GPNMB-sufficient DBA/2J mice (D2.*Gpnmb*^+^) were assessed as an age- and background-matched normotensive control [43].

**Fig. 2:**
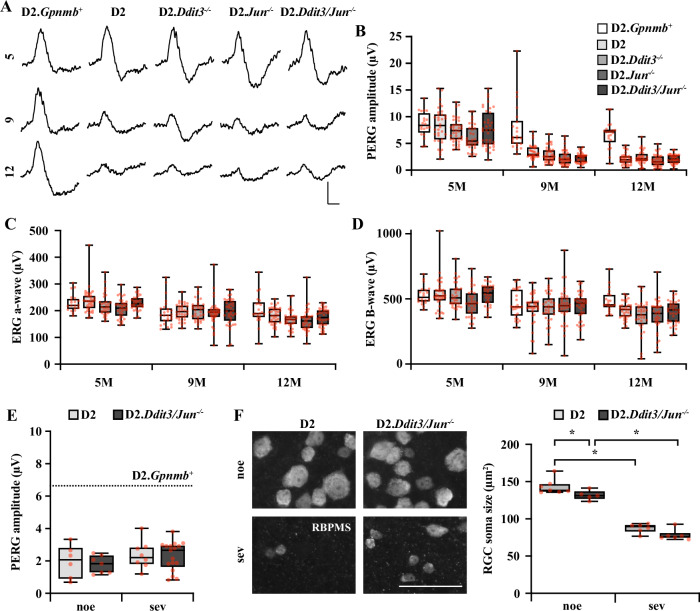
*Ddit3/Jun* deletion did not prevent decline of PERG amplitudes in DBA/2J mice. **A** Representative pattern electroretinography (PERG) traces and amplitude quantification (**B**) from D2.*Gpnmb*^+^, D2, D2.*Ddit3*^*−/−*^, D2.*Jun*^*−/−*^, and D2.*Ddit3/Jun*^−/−^ mice at 5 M (*n* = 20, 34, 38, 34, 39), 9 M (*n* = 22, 34, 38, 39, 36), and 12 M (*n* = 21, 34, 36, 35, 35). Normotensive D2.*Gpnmb*^+^ mice did not have significant decline in PERG amplitude at 9 M (*P* = 0.220), but had a slight but significant decline in PERG amplitude by 12 M compared to 5 M (*P = 0.001), Ocular hypertensive mice of all genotype groups had significant PERG amplitude decline at 9 M and 12 M compared to 5 M (*P < 0.001). At 9 M and 12 M, each ocular hypertensive group’s PERG amplitude was significantly lower than normotensive D2,*Gpnmb*^+^ controls (*P < 0.001). No increase in PERG amplitude was observed between D2 and D2.*Ddit3*^*−/−*^, D2.*Jun*^*−/−*^, or D2.*Ddit3/Jun*^*−/−*^ groups at any timepoint measured. Scale bar: Y: 5 μV, X: 100 ms. Mixed effects analysis, Holm-Sidak’s *post hoc*. **C** Quantification of full-field ERG a-wave and b-wave (**D**) amplitudes in D2.*Gpnmb*^+^, D2, D2.*Ddit3*^*−/−*^, D2.*Jun*^*−/−*^, and D2.*Ddit3/Jun*^*−/−*^ eyes. By 12 M (*n* = 21, 36, 35, 33, 37), each ocular hypertensive group had a slight but significant decline of electroretinography (ERG) a and b-wave amplitudes compared to 5 M (*n* = 20, 38, 30, 34, 30) (*P < 0.001), but not nearly to the same extent as PERG amplitude decline (**A**). Two-way ANOVA, Holm-Sidak’s *post hoc*. Percentage of PERG and ERG amplitude declines at 9 and 12 M are listed for each group in Table 1. **E** PERG amplitude quantifications from 12 M D2 and D2.*Ddit3/Jun*^*-/-*^ retinas with noe (*n* = 6, 7) and sev (*n* = 8, 17) optic nerve damage. Neither genotype nor optic nerve damage level influenced PERG amplitude (*P* > 0.05, Two-way ANOVA). Of note, PERG amplitudes were significantly reduced compared with 12 M D2.*Gpnmb*^+^ (*n* = 21, 7.2 ± 0.6) regardless of genotype or level of axonal damage (*P < 0.001, One-way ANOVA, Holm-Sidak’s *post hoc*). PERG amplitude (μV) ± SEM from D2 and D2.*Ddit3/Jun*^*−/−*^ retinas, respectively: noe: 2.0 ± 0.4, 1.8 ± 0.2; sev: 2.3 ± 0.3; 1.9 ± 0.2. Scale bar: Y: 5 μV, X: 100 ms. **F** High-resolution images of retinal flat mounts immunoassayed for RBPMS (scale bar, 50μm) and quantification of average RGC soma size from D2 and D2.*Ddit3/Jun*^*−/−*^ retinas with noe (*n* = 7, 5) and sev (*n* = 6, 6) glaucomatous damage. Both genotype groups had significant reductions in RGC soma size in sev glaucoma compared to respective noe controls (*P < 0.001). While D2.*Ddit3/Jun*^−/−^ noe retinas had slightly smaller RGCs (*P = 0.044), *Ddit3/Jun* deletion did not attenuate RGC soma shrinkage in sev retinas. Soma size (μm^2^) ± SEM from D2 and D2.*Ddit3/Jun*^*−/−*^ retinas, respectively: noe: 143.1 ± 3.9, 131.7 ± 2.9; sev: 87.6 ± 2.5, 78.3 ± 3.0. Two-way ANOVA, Holm-Sidak’s *post hoc*.

Compared to non-glaucomatous D2.*Gpnmb*^+^ age-matched controls, PERG amplitude significantly declined in all genotype groups over time (Fig. 2A, B). As previously observed, PERG amplitude significantly declined by 9 M for DBA/2J mice compared to D2.*Gpnmb*^+^ age-matched controls [51], and *Ddit3/Jun* deficiency did not prevent this decline. Similar results were observed at 12 M. Thus, despite conferring robust protection to RGC somas, *Ddit3*/*Jun* deletion did not prevent loss of RGC somal function. Notably, PERG amplitude decline did not appear to be caused by gross photoreceptor or bipolar cell dysfunction or improper light penetration. ERG a- and b-wave amplitudes declined slightly with age for all genotype groups, including D2.*Gpnmb*^+^, but not nearly to the same extent as PERG amplitude decline (Fig. 2C, D). %PERG and ERG a- and b-wave amplitudes relative to respective 5 M controls are listed in Table 1.

**Table 1:** Amplitudes of PERGs, ERG a-waves, and ERG b-waves expressed as a percentage of respective 5 M amplitudes.

		D2.*Gpnmb*^*+*^	D2	D2.*Ddit3*^*−/−*^	D2.*Jun*^*−/−*^	D2.*Ddit3/Jun*^*−/−*^
%PERG amplitude	9 M	89.0 ± 9.9	**39.8** ± 2.9*	**38.8** ± 3.1*	**38.3** ± 3.6*	**29.6** ± 2.1*
	12 M	**77.2** ± **6.3**	**25.0** ± 2.1*	**31.7** ± 2.8*	**29.7** ± 3.1*	**27.4** ± 1.9*
%ERG a wave amplitude	9 M	**84.0** ± **4.2**	**82.5** ± **2.2**	90.4 ± 2.9	92.0 ± 3.8	**86.0** ± **3.5**
	12 M	89.0 ± 5.3	**76.4** ± **2.2**	**75.5** ± 2.6*	**78.6** ± 3.6*	**76.4** ± 2.4*
%ERG b wave amplitude	9 M	**86.0** ± **3.9**	**79.7** ± **3.7**	**81.9** ± **3.6**	95.2 ± 4.7	**83.8** ± **3.2**
	12 M	91.5 ± 3.6	**76.8** ± 2.0*	**68.3** ± 4.1*	**79.2** ± 4.6*	**75.0** ± 2.7*

*Bold text:* *P* < 0.05 compared to respective 5 M.

*: *P* < 0.05 compared to age-matched D2.*Gpnmb*^+^.

Notably, PERG amplitude decline did not depend on morphologically observed RGC loss. Regardless of genotype, 12 M eyes with no or early and severe glaucomatous damage had similarly reduced PERG amplitudes relative to age-matched D2.*Gpnmb*^+^ controls (Fig. 2E). Interestingly, *Ddit3/Jun* deletion did not prevent ocular hypertension-induced shrinkage of the RGC soma (Fig. 2F), suggesting surviving RGCs are likely injured and/or undergoing metabolic stress [53, 54]. Thus, despite conferring protection from somal loss, *Ddit3/Jun* deletion did not preserve RGC somal viability, at least as measured by gross potentials and soma size. These data suggest the mechanism(s) driving somal shrinkage and loss of gross potentials must act either upstream or independently of somal DDIT3/JUN activation.

### MKK4 and MKK7 controlled RGC somal and axonal degeneration after axonal injury

MKK4 and MKK7 (MAP2Ks 4 and 7) are known to control DDIT3 and JUN activation after injury and thus may drive RGC somal death after axonal injury. In addition, MKK4/7 activate a variety of downstream targets independently of DDIT3/JUN activation, which may contribute to decline of somal viability and axonal degeneration. In fact, recent work has suggested the importance of both MKK4 and MKK7 in driving axonal degeneration [32, 38]. Therefore, MKK4/7 activation may drive degeneration of the entire RGC. To test this possibility, C57BL/6 J mice with homozygous *Mkk4*^*fl*^ and *Mkk7*^*fl*^ alleles (B6.*Mkk4*^*fl/fl*^*Mkk7*^*fl/fl*^) were treated with intravitreal AAV2.2-delivered Cmv-cre (AAV2.2-Cmv-cre-Gfp) to generate animals with *Mkk4/7*^*−/−*^ RGCs (B6.*Mkk4/7*^*−/−*^). AAV2.2 with no cre vector (AAV2.2-Cmv-Gfp) was intravitreally injected into B6.*Mkk4*^*?*^*Mkk7*^*?*^ eyes to generate WT B6.*Mkk4/7*^+/+^ controls.

**Fig. 3:**
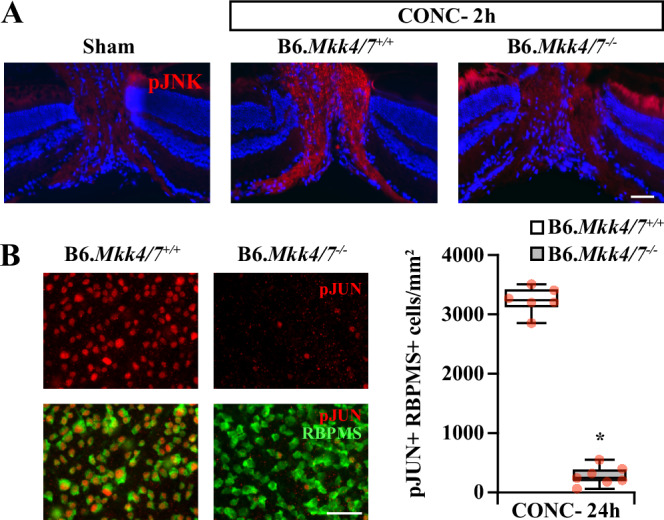
Efficient RGC recombination of *Mkk4* and *Mkk7* floxed alleles with AAV2.2 delivered Cmv-cre. **A** Representative sections of B6.*Mkk4/7*^*+/+*^ (*n* = 3) and B6.*Mkk4/7*^*−/−*^ (*n* = 3) optic nerve heads 2 h after CONC or Sham (*n* = 3) immunoassayed for activated JNK (pJNK). JNK was markedly activated in B6.*Mkk4/7*^*+/+*^ optic nerves, but not in B6.*Mkk4/7*^*−/−*^ optic nerves, 2 h after CONC. **B** Representative B6.*Mkk4/7*^*+/+*^ (*n* = 6) and B6.*Mkk4/7*^*−/−*^ (*n* = 7) retinal flat mounts 24 h after CONC immunoassayed for RBPMS (green) and activated JUN (pJUN, red) and quantification of pJUN+ RBPMS+ cells. RGC JUN activation was reduced by 91.4 ± 1.8% (*P < 0.001) in B6.*Mkk4/7*^*−/−*^ retinas. pJUN+RBPMS+ cells/mm^2^: B6.*Mkk4/7*^*+/+*^: 3239.6 ± 91.9; B6.*Mkk4/7*^*-/-*^: 278.0 ± 59.8. Two-tailed *t* test. Scale bar, 50 μm.

**Fig. 4:**
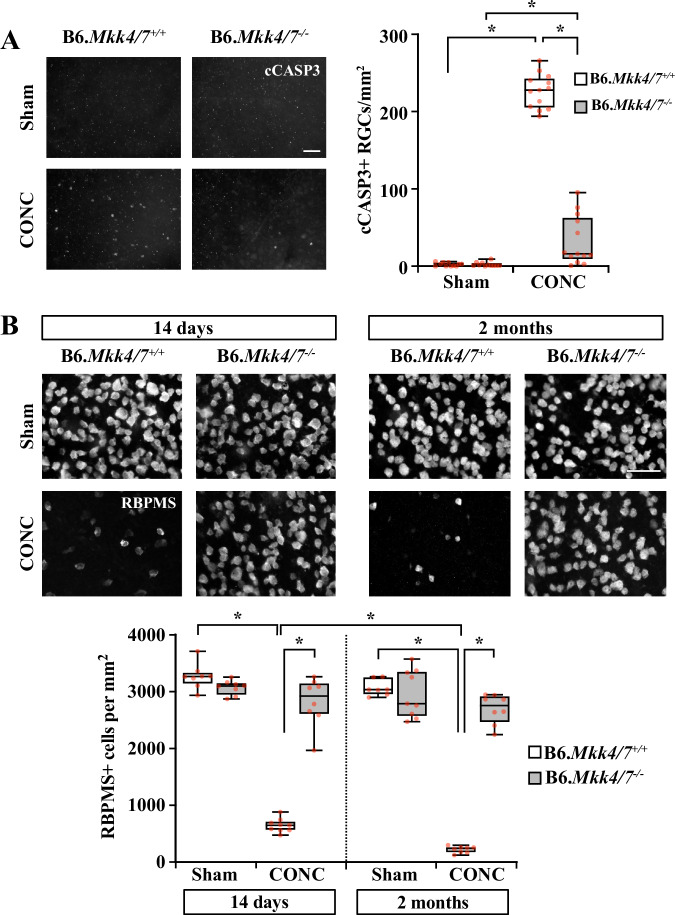
*Mkk4/7* deletion protected nearly all RGC somas after axonal injury. **A** Representative B6.*Mkk4/7*^*+/+*^ and B6.*Mkk4/7*^*-/-*^ retinal flat mounts 5 days after Sham (*n* = 11, 11) or CONC (*n* = 13, 13) procedures immunoassayed for activated caspase 3 (cCASP3). *Mkk4/7* deletion prevented 85.6 ± 3.9% (*P < 0.001) of caspase 3 activation. cCASP3+RGCs/mm^2^ ± SEM for B6.*Mkk4/7*^*+/+*^ and B6.*Mkk4/7*^*−/−*^ respectively: Sham: 2.2 ± 0.7, 2.4 ± 0.8; CONC: 226.5 ± 6.1, 32.5 ± 8.8. Two-way ANOVA, Holm-Sidak’s *post hoc*. **B** Representative B6.*Mkk4/7*^+/+^ and B6.*Mkk4/7*^−/−^ retinal flat mounts 14 days and 2 months after Sham or CONC immunoassayed for RBPMS and quantification of RBPMS+ cell survival. *Mkk4/7* deletion prevented 89.9 ± 6.1% and 90.2 ± 3.3% of RGC death at 14 days and 2 months, respectively. RBPMS+ cells/mm^2^ ± SEM and *n* for 14 days—B6.*Mkk4/7*^*+/+*^ Sham: 3264.7 ± 77.8 *n* = 8, B6.*Mkk4/7*^*−/−*^ Sham: 3067.8 ± 45.1 *n* = 8; B6.*Mkk4/7*^*+/+*^ CONC: 667.1 ± 38.0 *n* = 9; B6.*Mkk4/7*^*−/−*^ CONC: 2819.0 ± 149.6 *n* = 8. For 2 months—B6.*Mkk4/7*^*+/+*^ Sham: 3063.0 ± 53.2 *n* = 7; B6.*Mkk4/7*^*−/−*^ Sham: 3015.6 ± 144.6 *n* = 9; B6.*Mkk4/7*^*+/+*^ CONC: 222.6 ± 21.0 *n* = 8; B6.*Mkk4/7*^*−/−*^ CONC: 2690.7 ± 91.7 *n* = 8. (B6.*Mkk4/7*^*+/+*^ CONC 14 days vs 2 months, *P < 0.05; B6.*Mkk4/7*^*+/+*^ Sham vs CONC at 14 days and 2 months, *P < 0.001). Three-way ANOVA, Holm-Sidak’s *post hoc*. Scale bars, 50 μm.

To ensure sufficient recombination of *Mkk4/7* floxed alleles, activation of MKK4/7’s downstream targets were evaluated after CONC. Robust JNK activation in the optic nerve head and JUN activation in RGC somas occurs early after CONC [18, 21]. Compared to B6.*Mkk4/7*^*+/+*^ controls, B6.*Mkk4/7*^*−/−*^ eyes had little appreciable JNK activation in the optic nerve head (Fig. 3A) and had a 91% reduction of RGCs with JUN activation (Fig. 3B) after CONC. Therefore, AAV2.2-delivered Cmv-cre effectively recombined *Mkk4/7* floxed alleles in RGCs. To determine whether MKK4 and MKK7 together play an important role in RGC somal loss after glaucoma-relevant injury, RGC soma survival was assessed for B6.*Mkk4/7*^+/+^ and B6.*Mkk4/7*^−/−^ retinas after CONC. *Mkk4/7* deletion provided robust and sustained long-term protection to RGC somas after CONC—*Mkk4/7* deletion prevented the vast majority of caspase 3 activation 5 days post-CONC (Fig. 4A) and preserved 90% of RGC somas at both 14 days and 2 months post-CONC (Fig. 4B). Therefore, MKK4 and MKK7 controlled RGC somal death after glaucoma-relevant injury.

**Fig. 5:**
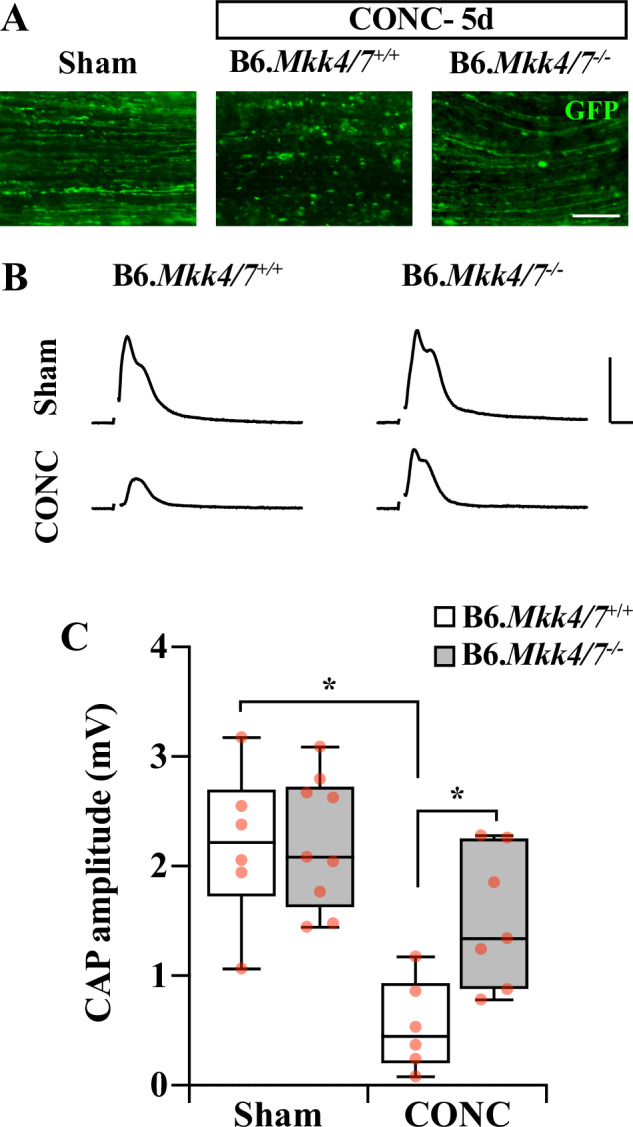
*Mkk4/7* deletion prevented RGC axonal degeneration after axonal injury. **A** Representative longitudinal sections of B6.*Mkk4/7*^*+/+*^ and B6.*Mkk4/7*^*-/-*^ optic nerves 5 days after Sham (*n* = 6, 9) or CONC (*n* = 6, 7) procedures. B6.*Mkk4/7*^*−/−*^ optic nerves had improved axonal integrity compared to B6.*Mkk4/7*^*+/+*^ controls. Note: AAV2.2-CMV-Cre-GFP and AAV2.2-CMV-GFP controls both induce GFP expression, therefore RGC axons were identified with GFP. Scale bar, 50 μm. **B** Representative compound action potential (CAP) traces and amplitude quantifications (**C**) from of B6.*Mkk4/7*^*+/+*^ and B6.*Mkk4/7*^*−/−*^ optic nerves 5 days after CONC or Sham procedures. *Mkk4/7* deletion provided significant protection from CAP amplitude decline after CONC. CAP amplitude (mV) ± SEM from B6.*Mkk4/7*^*+/+*^ and B6.*Mkk4/7*^*−/−*^, respectively: Sham: 2.8 ± 0.3, 2.8 ± 0.2; CONC: 0.9 ± 0.2, 2.0 ± 0.3. (B6.*Mkk4/7*^*+/+*^ Sham vs B6.*Mkk4/7*^*+/+*^ CONC: *P < 0.001; B6.*Mkk4/7*^*+/+*^ CONC vs B6.*Mkk4/7*^*−/−*^ CONC: *P = 0.007; B6.*Mkk4/7*^*−/−*^ Sham vs B6.*Mkk4/7*^*−/−*^ CONC: *P = 0.028.) Two-way ANOVA, Holm-Sidak’s *post hoc*. Scale bar: Y: 2 mV, X: 1 ms.

Recent evidence has suggested a role for MKK4/7 in driving Wallerian degeneration cascades after glaucoma-relevant injury [32, 38]. To clarify the role of MKK4/7 in axonal degeneration, RGC axonal integrity was evaluated for B6.*Mkk4/7*^*+/+*^ and B6.*Mkk4/7*^−/−^ optic nerves after glaucoma-relevant injury. Consistent with previous reports [32, 38], B6.*Mkk4/7*^*−/−*^ RGC axons had substantially fewer histological indications of degeneration (Fig. 5A). Importantly, *Mkk4/7* deletion preserved axonal physiological function as assessed by compound action potentials (CAPs, Fig. 5B, C). These data show that together, MKK4 and MKK7 control degeneration of both the RGC soma and axon after axonal injury.

**Fig. 6:**
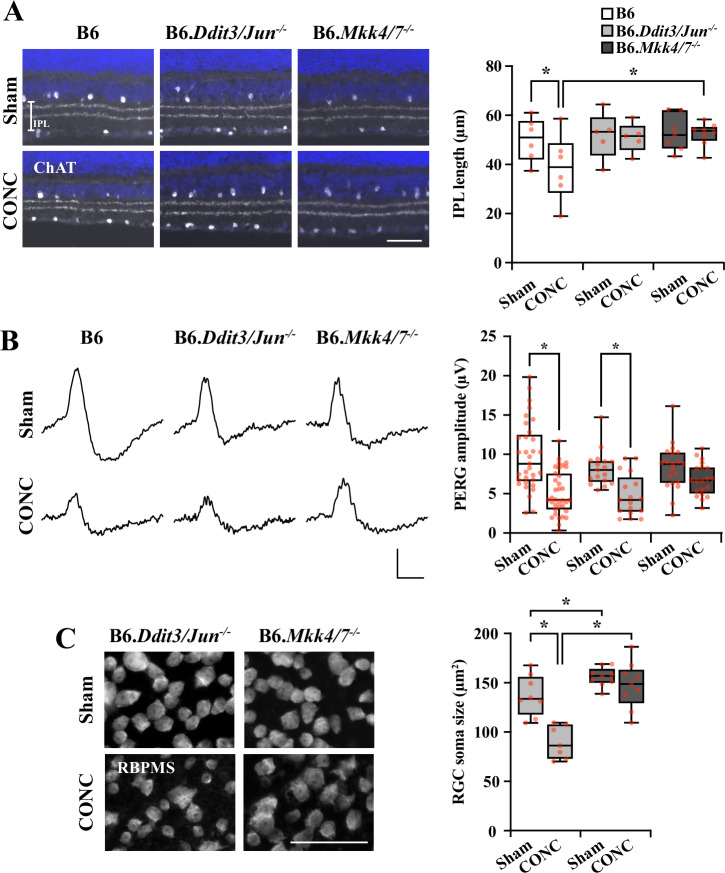
*Mkk4/7* deletion, but not *Ddit3/Jun* deletion, prevented PERG amplitude decline and somal shrinkage after axonal injury. **A** Representative sections and quantification of IPL length from B6, B6.*Ddit3/Jun*^*−/−*^, and B6.*Mkk4/7*^*−/−*^ retinas 35 days post-Sham (*n* = 6, 5, 7) or CONC (*n* = 6, 5, 8) procedures. B6 IPL length significantly decreased 35 days post-CONC (by 22.7 ± 11%, *P = 0.030), while B6.*Ddit3/Jun*^*−/−*^ and B6.*Mkk4/7*^*−/−*^ IPL lengths did not significantly change after CONC (*P* = 0.883 and *P* = 0.925, respectively). B6.*Mkk4/7*^*−/−*^ IPLs were significantly longer than B6 IPLs post-CONC (*P = 0.020). Note, in this experiment, *Jun*^*fl*^ alleles were recombined from the retina using bilateral intravitreal delivery of AAV2.2-CMV-Cre-GFP. IPL length (µm) ± SEM for B6, B6.*Ddit3/Jun*^*−/−*^ and B6.*Mkk4/7*^*−/−*^ mice, respectively: Sham: 50.1 ± 3.6, 51.8 ± 4.3, 52.8 ± 2.8; CONC: 38.7 ± 5.5, 51.0 ± 2.7, 52.4 ± 1.7. Two-way ANOVA, Holm-Sidak’s *post hoc*. Scale bar, 50 μm. **B** Representative PERG traces and quantification of PERG amplitudes from B6, B6.*Ddit3/Jun*^*−/−*^, and B6.*Mkk4/7*^*−/−*^ retinas 14 days post- Sham (*n* = 33, 17, 20) or CONC (*n* = 35, 17, 20) procedures. B6 and B6.*Ddit3/Jun*^*−/−*^ PERG amplitudes significantly declined 14 days post-CONC (by 52.6 ± 5.7%, *P < 0.001 and 57.9 ± 7.9%, *P = 0.002, respectively), while B6.*Mkk4/7*^*−/−*^ PERG amplitudes did not significantly decline (*P* = 0.108). Two-way ANOVA, Holm-Sidak’s *post-hoc*. Scale bar: Y: 5 μV, X: 100 ms. **C** Representative high-resolution images of retinal flat mounts immunoassayed for RBPMS and quantification of RGC soma size from B6.*Ddit3/Jun*^−/−^ and B6.*Mkk4/7*^*−/−*^ retinas 14 days post-Sham (*n* = 8, 9) or CONC (*n* = 7, 9) procedures. After CONC, B6.*Ddit3/Jun*^*−/−*^ RGC somas were reduced to 65.5 ± 4.6% (*P < 0.001) the size of respective RGCs after Sham procedures. B6.*Mkk4/7*^*−/−*^ RGC soma sizes did not change after CONC compared to Sham (*P* = 0.439). B6.*Mkk4/7*^*−/−*^ RGCs were significantly larger compared to B6.*Ddit3/Jun*^*−/−*^ (*P < 0.001) RGCs after CONC. Soma size (μm^2^) ± SEM from B6.*Ddit3/Jun*^*−/−*^ and B6.*Mkk4/7*^*−/−*^ mice, respectively: Sham: 137.0 ± 7.3, 155.8 ± 3.2; CONC: 89.7 ± 6.3, 147.5 ± 7.8. Two-way ANOVA, Holm-Sidak’s *post hoc*. Scale bar, 50 μm.

Given DBA/2J PERG amplitude decline and soma shrinkage were not prevented with *Ddit3/Jun* deletion despite robust somal survival, it remained important to assess RGC somal function and size in B6.*Mkk4/7*^*−/−*^ eyes after glaucoma relevant injury. Consistent with patterns of RGC soma survival, both *Ddit3/Jun* and *Mkk4*/*7* deletion from C57BL/5J RGCs prevented thinning of the inner plexiform layer (IPL) after CONC (Fig. 6A). However, in striking contrast to *Ddit3/Jun* deletion, *Mkk4*/*7* deletion significantly attenuated PERG amplitude decline (Fig. 6B) and soma shrinkage (Fig. 6C) after CONC. These data suggest MKK4/7 govern not only somal and axonal survival, but also drive loss of some aspects of viability and gross function. Thus, activation of MKK4/7 is likely a critical inciting event integrating mechanisms controlling death and degeneration of somal and axonal RGC compartments in the context of axonal injury.

## Discussion

The present study investigated the degenerative mechanisms by which RGCs die after glaucoma-relevant injury. Specifically, we examined the dual role of DDIT3 and JUN and their upstream regulators MKK4/7 in controlling RGC death after glaucoma-relevant injury. DDIT3/JUN controlled the majority of RGC somal death in ocular hypertensive DBA/2 J mice but did not control axonal degeneration. Despite playing a critical role in RGC somal loss, DDIT3/JUN did not contribute to decline in somal viability as measured by RGC somal shrinkage and loss of PERG amplitudes in DBA/2 J mice or after CONC. In contrast, *Mkk4/7* deficiency significantly lessened not only somal apoptosis but also PERG amplitude decline, somal shrinkage, and the rate of axonal degeneration after glaucoma-relevant axonal injury. Together, these data suggest activation of MKK4/7 is a critical event after glaucoma-relevant injury which activates pathways governing degeneration of RGC axons and somas. Future studies should assess the importance of MKK4/7 in driving RGC degeneration after chronic ocular hypertension. Furthermore, identifying the upstream activators of MKK4/7 in the context of glaucoma-relevant injury and elucidating the DDIT3/JUN-independent downstream effectors of MKK4/7 driving loss of somal viability and axonal degeneration will be important next lines of investigation.

Upstream regulators of MKK4 and MKK7 have previously been implicated in driving axonal and somal degenerative cascades after glaucoma relevant injury. Several studies have suggested the importance of Dual leucine kinase (DLK, MAP3K12), an upstream activator of MKK4 and MKK7, in initiating cell death pathways important in somal and axonal degeneration [12, 55, 56]. Inhibition of DLK lessened Wallerian degeneration after axonal injury in vitro [37, 38, 56] and in vivo [38, 56]. DLK inhibition modestly protected RGC somas and axons in a model of inducible ocular hypertension [57]. However, deletion of *Dlk* did not phenocopy *Mkk4/7* deletion’s protection to RGC axons after CONC [12]. Importantly, *Dlk* deletion was not sufficient to prevent axonal JNK activation after CONC [12], suggesting *Dlk* is not the sole activator of MKK4/7 after mechanical optic nerve injury. For example, MKK4/7 are also known to be activated by the MAP3K LZK [58, 59]. Thus, it is possible that multiple MAP3Ks contribute to MKK4/7 activation in the context of glaucoma-relevant injury. Regardless of their upstream activators, MKK4/7 together controlled death of somal and axonal compartments after axonal injury. Our findings suggest MKK4/7 drive somal loss, possibly via activation of DDIT3 and JUN. However, axonal degeneration, somal PERG decline, and soma shrinkage are likely be driven by MKK4/7 via a mechanism independent of DDIT3/JUN activation.

Much research has begun to uncover the mechanisms important in governing Wallerian axonal degeneration—many of which may be controlled by MKK4/7 after glaucoma-relevant injury. For example, recent work has suggested the importance of JNK signaling in axonal degeneration. Loss of JNK1, 2, and 3 activation prevented axonal degeneration in vitro [32, 38, 56] and in vivo after axonal injury [56, 60], including after CONC [38], suggesting MKK4/7 mediate axonal degeneration via activation of the JNKs. Importantly, although JNK2/3 are the JNKs known to be expressed in the central nervous system, *Jnk2/3* deletion did not prevent axonal degeneration after CONC [12] or in DBA/2J mice [17]. These data either indicate a critical role for JNK1 in mediating axonal degeneration or suggest MKK4/7 mediate axonal degeneration through mechanisms in addition to or independently of JNK activation.

MKK4/7 (potentially via JNK activation) may drive axonal degeneration by contributing to NMNAT2 depletion after axonal injury. Walker et al. showed *Mkk4/7* silencing prevented NMNAT2 degradation and subsequent axonal degeneration in cultured dorsal root ganglion cells after axotomy. Furthermore, this study showed MKK4/7 activation and consequential NMNAT2 degradation acted upstream of pro-degenerative SARM1 activation to drive axonal degeneration [32] (NMNAT2 degradation has been shown to act upstream of SARM1 activation in several models of axon injury [32, 37, 61–63]). It has been suggested members of the MAPK cascade, including DLK, directly target NMNAT2 for degradation [36, 37]. It is possible MKK4/7 and/or JNK1/2/3 directly target NMNAT2 for degradation, ultimately triggering SARM1 activation and allowing axonal degeneration after glaucoma-relevant injury. Alternatively, work done by Yang et al. suggested SARM1 activity and NMNAT2 depletion act upstream of MKK4/7-JNK1/2/3 activation to drive Wallerian degeneration after axonal injury [38]. However, recent work has shown *Sarm1* deletion does not phenocopy *Mkk4/7* deletion after CONC; while *Sarm1* deletion protected RGC axons, it did not prevent somal JUN activation and subsequent somal death [13]. Thus, it remains more likely MKK4/7 drive axonal degeneration by facilitating NMNAT2 degradation and allowing pro-degenerative SARM1 activation. Future work should elucidate the mechanisms downstream of MKK4/7 activation that ultimately drive Wallerian degeneration in the context of glaucomatous injury.

An intriguing result of our study indicated glaucoma-relevant loss of RGC gross potentials and soma shrinkage are not merely consequences of somal loss. To date, few studies have investigated the compartment-specific RGC-intrinsic mechanisms by which RGCs undergo shrinkage and lose the ability to fire in response to light-evoked retinal neuronal signals. Interestingly, previous work suggested *Bax* deletion, despite providing protection against neuronal loss, did not prevent somal shrinkage after neurotrophic deprivation. In this study, soma shrinkage corresponded with indicators of metabolic stress such as loss of glucose uptake and decreased rate of protein synthesis—which were not prevented by *Bax* deletion [54]. In contrast, inhibition of mixed lineage kinases (and downstream MKK4/7 and JNK signaling) preserved soma size and metabolic integrity after trophic deprivation [53]. Consistent with our reported results, these data indicate MKK4/7 promote deterioration of aspects of somal health independently of DDIT3/JUN-induced BAX activation.

It is possible MKK4/7 mediate somal and axonal decline via DDIT3/JUN-independent NMNAT2 degradation. WLD^S^ and *Nmnat* gene therapy’s preservation of RGC electrical function in ocular hypertensive mice suggests a role for NMNAT2 in maintaining RGC somal viability [3, 48, 64, 65]. It is also important to consider manipulations like WLD^S^ and *Nmnat1* gene therapy preserved both RGC somas and axons in DBA/2J mice [3, 48, 49, 66]. This could suggest protection of both the RGC soma and axon is required for protection of somal viability after glaucoma-relevant injury. Work by Chou et al. showed temporarily blocking RGC retrograde axonal transport without injuring RGCs caused a significant and reversable reduction in PERG amplitude [67]. Thus, loss of axonal transport itself could lead to subsequent loss of somal gross potentials, suggesting preservation of axons (and therefore preservation of axon transport) by *Mkk4/7* deletion could also preserve RGC somal gross potentials.

MKK4/7 could also drive RGC degeneration via activation of DDIT3/JUN-independent mechanisms in the soma. For example, MKK4/7 may drive somal and axonal degeneration via activation of pro-apoptotic BH3-only proteins. JNK is known to phosphorylate and activate pro-apoptotic BH3-only proteins, thereby inhibiting pro-survival Bcl-2 family proteins and allowing BAX activation [34]. Overexpression of *Bcl2l1* (*Bclxl*) prevented axonal degeneration in DBA/2J mice [68] and reduced RGC soma loss but not axonal degeneration after axonal injury [19, 69]. Notably, *Bclxl* overexpression did not prevent somal shrinkage or PERG amplitude decline after axonal injury, suggesting BCLXL loss is not the sole driver of RGC somal degeneration in the context of glaucoma. *Bcl2* overexpression also protected RGC somas [70–72] but not distal axons [71, 72] after CONC. Interestingly, *Bcl2* overexpression prevented PERG amplitude decline even up to 2 months post-CONC [50], suggesting a potential role of Bcl2 family proteins in maintaining loss of somal viability after injury. Therefore, BCL2 or other Bcl-2 family proteins may be critical in maintaining somal survival, somal health, and axonal degeneration, and MKK4/7-mediated inhibition of Bcl-2 family proteins may contribute to RGC degeneration in glaucoma.

In conclusion, we demonstrate MKK4/7 controlled RGC soma loss after glaucoma-relevant injury—possibly via somal DDIT3/JUN activation. MKK4/7 also controlled RGC axonal degeneration and decline PERG amplitude and somal size via DDIT3/JUN-independent mechanisms. These data suggest MKK4/7 activation may be an inciting mechanism initiating somal and axonal degenerative cascades in glaucoma. The DDIT3/JUN-independent mechanisms by which MKK4/7 drive axonal degeneration cascades and deterioration of somal health remain unidentified. Future work should elucidate the downstream effectors of MKK4/7 in each RGC compartment and should also identify upstream activators of MKK4/7 in the context of glaucoma to further identify neurotherapeutic targets.

## Methods

### Mice

*Ddit3* null alleles [73] (Jackson Laboratory, Stock# 005530), floxed alleles of *Jun* [74] (*Jun*^*fl*^), and the Six3-cre transgene [75] (Jackson Laboratory, Stock# 019755) were backcrossed >10 times to both the C57BL/6 J genetic background (>99% C57BL/6J) and the DBA/2J background (>99% DBA/2J). *Jun*^*fl*^ alleles were recombined in the optic cup using Six3-cre, except for one experiment (Fig. 6A) where *Jun*^*fl*^ alleles were recombined from retinal neurons via bilateral intravitreal delivery of AAV2.2-CMV-Cre-GFP (UNC vector core; AAV2.2-CMV-GFP with no cre was injected as a control). Floxed alleles of *Mkk4* [76] and *Mkk7* [77] were backcrossed >10 times to the C57BL/6J background and were recombined from retinal neurons via bilateral intravitreal delivery of AAV2.2-CMV-Cre-GFP (UNC vector core; AAV2.2-CMV-GFP with no cre was injected as a control). Mice were fed chow and water ad libitum and were housed on a 12-h light-to-dark cycle. All experiments were conducted in adherence with the Association for Research in Vision and Ophthalmology’s statement on the use of animals in ophthalmic and vision research and were approved by the University of Rochester’s University Committee on Animal Resources (UCAR-2006-085E).

#### Experimental rigor and statistical analysis

For all procedures, the experimenter was masked to genotype and condition. Roughly equal numbers of male and female mice were used for each experimental group. B6 mice were 3–6 M of age, while D2 mice were aged as indicated. Animals were randomly assigned to experimental groups. Before experiments were performed, it was established that animals with pre-existing abnormal eye phenotypes (e.g. displaced pupil, cataracts) would be excluded from the study.

Data were analyzed using GraphPad Prism9 software. Power calculations were performed before experiments were conducted to determine appropriate sample size. The comparison of the percent of optic nerves at each grade between genotypes was analyzed using a Chi-square test. Data from experiments designed to test differences between two groups were subjected to an F test to compare variance and a Shapiro-Wilk test to test normality to ensure appropriate statistical tests were utilized. For normally distributed data with equal variance, a two-tailed independent samples *t* test was utilized. Data from experiments designed to test differences among more than two groups across one condition were subjected to a Brown-Forsythe test to compare variance and a Shapiro-Wilk test to test normality to ensure an appropriate statistical test was utilized. Normally distributed data with equal variance were analyzed using a one-way ANOVA followed by Holm-Sidak’s post-hoc test. Non-normally distributed data were analyzed using a Kruskal-Wallis test with Dunn’s post-hoc test. Data from experiments designed to detect differences among multiple groups and across two conditions were analyzed using a two-way ANOVA followed by Holm-Sidak’s post-hoc test. Data from experiments designed to test differences among multiple groups across more than two conditions were analyzed using a three-way ANOVA followed by Holm-Sidak’s post-hoc test. For these statistical tests, every possible comparison was made when relevant, and multiplicity adjusted *P* values are reported. In all cases, data met the assumptions of the statistical test used. *P* values < 0.05 were considered statistically significant. Throughout the manuscript, results are reported as mean ± standard error of the mean (SEM).

#### Full field and pattern electroretinograms

Pattern and full-field electroretinography (PERGs and ERGs, respectively) was conducted using Diagnosys LLC’s Celeris rodent ERG system according to manufacturer’s instructions. Briefly, mice were dark-adapted for 60 minutes, as described previously [78, 79]. Mice were anaesthetized with an intraperitoneal injection of 0.05 ml/10 g solution containing 20 mg/mL ketamine and 2 mg/mL xylazine. Hypromellose GenTeal (0.3%, Novartis Pharmaceuticals Corporation, NDC 0078-0429-47) was applied to the eyes before placement of electrodes. PERGs were obtained using 50 cd/m^2^ mean luminance with spatial frequency 0.155 cycles/degree with 100% contrast. A total of 600 sweeps were recorded and averaged per eye. ERGs were obtained with 1 cd s/m^2^ luminance. For ERG and PERG, corneal electrodes were carefully placed at consistent angles according to manufacturer’s instructions.

#### Other surgeries and procedures

Mice were anaesthetized with an intraperitoneal injection of 0.05 ml/10 g solution containing ketamine (20 mg/mL) and xylazine (2 mg/mL). Controlled optic nerve crush (CONC) [9, 39, 46] and intraocular pressure measurement [14, 46, 47] was performed as previously described. For intraviteal injections, the conjunctiva at the temporal quadrant was cleared away with the bevel of a 30-gauge needle, and a small incision was made with the 30-gauge needle through the sclera and behind the limbus. A Hamilton syringe (Hamilton Company, 7633–01) with a blunt 33-gauge needle was used to perform intravitreal injections. The needle of the Hamilton syringe was inserted 1 mm into the incision site at a 45° angle toward the optic nerve and was held in place for 30 s prior to injection. Over the course of 2 min, 1 μL of AAV2.2-CMV-Cre-GFP or AAV2.2-CMV-GFP (UNC vector core) was injected. The needle was held in place for 30 s following the injection and was removed over the course of 30 s. Antibiotic ointment was placed over the eye. CONC or Sham procedures were performed at least 28 days after intravitreal delivery of AAV2.2-Cmv-cre-gfp or AAV2.2-Cmv-gfp to allow for recombination and endogenous protein degradation.

#### Compound action potentials

Compound action potentials were recorded as previously described [12, 13, 21]. 5 days following CONC, animals were euthanized with CO_2_ asphyxiation and optic nerves were dissected free. Fresh optic nerves were transferred to a chamber of artificial cerebral spinal fluid (ACSF) aerated with 95% O_2_/5%CO_2_ for at least 60 minutes before recording. Optic nerves were transferred to a temperature-controlled chamber perfused with ACSF bubbled with 95% O_2_/5% CO_2_. Nerves were drawn into glass pipet suction electrodes (filled with ACSF) at each end for stimulation and recording. The recording pipet resistance was measured before (17–20 KΩ) and after (29–34 KΩ) insertion of the nerve and monitored continuously during the experiment. The ratio of this resistance during each sweep divided by the resistance of the pipet alone allowed a normalization of the amplitude of the compound action potential (CAP) to our standard ratio of 1.7 [12, 80]. This corrects for any drift in the seal resistance during an experiment. Signals were fed to one input of an AC differential amplifier of our design. The second input came from a pipet electrode placed near the recording electrode. This served to subtract much of the stimulus artifact. Stimuli of 50μs duration were delivered by an optically isolated constant current unit (WPI, Sarasota FL) driven by the computer. Stimulus currents were monitored by a linear optically coupled amplifier of our design. All signals were electronically low pass filtered with a cutoff of 10 kHz. All records were taken at 37 ± 0.5 °C.

#### Histology, nerve grading, and immunofluorescence

Optic nerve processing for plastic sectioning and optic nerve severity grading [14, 22] were performed as previously described. Immunofluorescence of whole-mounted and cryosectioned retinas [22, 39, 46] and cryosectioned optic nerves [12, 13] were performed as previously described using the following antibodies: rabbit anti-cCASP3 (AF835, R&D, 1:1000), rabbit anti-RBPMS (GTX118619, GeneTex, 1:250), chicken anti-GFP (ab13970-100, Abcam, 1:1000), rabbit anti-pJNK (4668S, Cell Signaling, 1:250), rabbit anti-pJUN (3270S, Cell Signaling, 1:250), goat anti-choline acetyltransferase (AB144P, Millipore-Sigma, 1:500), donkey anti-rabbit (A31572 and A-21206, ThermoFisher, 1:1000), donkey anti-mouse (A31570, ThermoFisher, 1:1000), donkey anti-goat (A21447, Invitrogen, 1:1000), and donkey anti-chicken (703-545-155, Jackson ImmunoResearch, 1:1000). Cryosections were counterstained with 4′,6-diamidino-2-phenylindole (DAPI). RGC soma sizes (measured using images assessed for RGC soma survival) were quantified using ImageJ by using a Gaussian blur filter with a sigma of 4, converting images to binary with an automatic triangle thresholding setting allowing detection of RGC somas. Somas were separated using the watershed function. Somas were defined as an area ≥30 µm^2^ and ≥0.2 circularity. RGCs cut off at the boarder of the image were excluded from analysis. Average soma area per image was measured, and 8 images were averaged per retina. Inner plexiform layer (IPL) length was measured in ImageJ from the lower edge of the inner nuclear layer and the upper edge of the ganglion cell layer, as indicated by DAPI and Choline acetyltransferase+ (ChAT+) amacrine cells. Images from three sections were taken at 10× at approximately 1000 µm from the peripheral edge of the section.

## Data Availability

The datasets used in the current study are available from the corresponding author on reasonable request.
